# Refined Wirtinger-type integral inequality

**DOI:** 10.1186/s13660-018-1700-4

**Published:** 2018-05-09

**Authors:** Liansheng Zhang, Shuxia Wang

**Affiliations:** 1 Department of Mathematics and Physics, Beijing Institute of Petro-Chemical Technology, Beijing, China; 2 Key Laboratory of Dependable Service Computing in Cyber Physical Society, Ministry of Education, Chongqing University, Chongqing, China

**Keywords:** Wirtinger-type double integral inequality, Stability analysis, Systems with time-delays

## Abstract

Based on the extreme value conditions of a multiple variables function, a new class of Wirtinger-type double integral inequality is established in this paper. The proposed inequality generalizes and refines the classical Wirtinger-based integral inequality and has less conservatism in comparison with Jensen’s double integral inequality and other double integral inequalities in the literature. Thus, the stability criteria for delayed control systems derived by the proposed refined Wirtinger-type integral inequality are less conservative than existing results in the literature.

## Introduction

During the past several decades, the Wirtinger inequality received considerable attention due to its extensive applications. For details, see [1–5] and the references therein. Recently, a Wirtinger-based integral inequality was presented by Seuret and Gouaisbaut in [1], and it was applied to analyze the stability for delayed systems. The Wirtinger-based inequality can deliver more accurate lower bounds for some single integral form of the quadratic terms which emerge in the derivative of Lyapunov–Krasovskii functional (LKF) (for example, $\int_{a}^{b} \omega^{T}(s)R_{1}\omega (s) \,ds$ ($R_{1} = R_{1}^{T} > 0$)) than other integral inequalities, such as the well-known Jensen inequality [6], Park’s inequality [7] and Moon’s inequality [8]. Accordingly, it is natural that the stability criterion derived by the Wirtinger-based integral inequality has less conservatism than those by the aforementioned inequalities.

On the other hand, based on Lyapunov stability theory, Sun et al. [9] firstly introduced the LKF containing the triple integral terms to discuss the stability problem. It was proved that this triple integral-based LKF can effectively reduce the conservatism of the stability criteria. Subsequently many researchers adopted this triple integral type LKF to analyze the stability for various delayed systems. As we know, when one chooses such LKF, such as $V = \int_{a}^{b} \,ds\int_{a}^{s} \,dr\int_{a}^{r} \omega^{T}(u)R_{2}\omega (u) \,du $ ($R_{2} = R_{2}^{T} > 0$), then some double integral terms, like $\int_{a}^{b} \,ds\int_{a}^{s} \omega^{T}(u)R_{2}\omega (u) \,du $ ($R_{2} = R_{2}^{T} > 0$) will emerge in the derivative of the chosen *V*. However, the previous Wirtinger-based integral inequality [1] cannot be applied in finding the lower bounds of such double integral terms since it merely evaluates the quadratic terms in the form of a single integral. It should be pointed out that, for such double integral terms, no other integral inequalities can handle them except Jensen’s double integral inequality [9, 10] at present. Nevertheless, Jensen’s double integral inequality is considerably conservative. This motivates our interest in reinvestigating this problem further.

In this paper, inspired by the method utilized in [2], we develop a new class of Wirtinger-type double integral inequality, as an extension of the Wirtinger-based integral inequality. In addition, Jensen’s double integral inequality can be regarded as a specialization of the newly derived Wirtinger-type double integral inequality.

The following notations are used throughout the paper. The superscript “*T*” denotes the transpose of a matrix. $\Re^{n}$ stands for the set of real vectors with dimension *n*. $R > 0$ denotes a symmetric positive definite matrix and $R < 0$ denotes a symmetric negative definite matrix. In a symmetric matrix, the symbol “*” is used to denote the term that is induced by symmetry. The symbol ⊗ stands for the Kronecker product. In addition, matrices whose dimensions are not explicitly stated are assumed to be compatible for algebraic operations.

## Main results

In this section, we are to derive the Wirtinger-type double integral inequality and its corollary.

### Theorem

(Wirtinger-type double integral inequality)

*If for a given symmetric matrix* $R > 0$, *two scalars* *a*, *b* *satisfy* $a < b$ *and we have a vector*-*valued function* $\omega (t):[a,b] \to \Re^{n}$ *such that the following integrations are well defined*, *then the following inequalities hold*:

1 $$\begin{aligned}& \frac{(b - a)^{2}}{2} \int_{a}^{b} \,ds \int_{a}^{s} \omega^{T}(u)R\omega (u) \,du \ge 3\xi^{T}\left ( \left [ \textstyle\begin{array}{c@{\quad}c} 3 & - 8 \\ * & 24 \end{array}\displaystyle \right ] \otimes R \right )\xi, \end{aligned}$$

2 $$\begin{aligned}& \frac{(b - a)^{2}}{2} \int_{a}^{b} \,ds \int_{s}^{b} \omega^{T}(u)R\omega (u) \,du \ge 3\eta^{T}\left ( \left [ \textstyle\begin{array}{c@{\quad}c} 3 & - 8 \\ * & 24 \end{array}\displaystyle \right ] \otimes R \right )\eta, \end{aligned}$$

*where*

$$\begin{gathered} \xi = \left [ \textstyle\begin{array}{c} \int_{a}^{b} \,ds \int_{a}^{s} \omega (u) \,du \\ \int_{a}^{b} \,ds\int_{a}^{s} \,dr \int_{a}^{r} \omega (u) \,du \end{array}\displaystyle \right ],\\ \eta = \left [ \textstyle\begin{array}{c} \int_{a}^{b} \,ds \int_{s}^{b} \omega (u) \,du \\ \int_{a}^{b} \,ds\int_{s}^{b} \,dr \int_{r}^{b} \omega (u) \,du \end{array}\displaystyle \right ].\end{gathered} $$

### Proof

Firstly, we are to prove inequality (1).

For a vector-valued function $\omega (t):[a,b] \to \Re^{n}$ satisfying the condition of the theorem, we define a function $z(u)$ as follows:

$$z(u) \triangleq \omega (u) - \frac{2}{(b - a)^{2}} \int_{a}^{b} \,ds \int_{a}^{s} \omega (r) \,dr - p(u)v, $$

where $p(u)$ is a scalar function and the constant vector *v* is to be determined.

Based on $z(u)$ defined above, we construct a generalized energy function $J(v)$ with respect to *v* denoted by

$$J(v) \triangleq \int_{a}^{b} \,ds \int_{a}^{s} z^{T}(u)Rz(u) \,du. $$

Then

$$\begin{aligned} \int_{a}^{b} \,ds \int_{a}^{s} z^{T}(u)Rz(u) \,du ={}& \int_{a}^{b} \,ds \int_{a}^{s} \omega^{T}(u)R\omega (u) \,du + \biggl( \int_{a}^{b} \,ds \int_{a}^{s} p^{2}(u) \,du \biggr) v^{T}Rv \\ &+ \frac{2}{(b - a)^{2}} \biggl( \int_{a}^{b} \,ds \int_{a}^{s} \omega (u) \,du \biggr)^{T}R \biggl( \int_{a}^{b} \,ds \int_{a}^{s} \omega (u) \,du \biggr) \\ &- \frac{4}{(b - a)^{2}} \biggl( \int_{a}^{b} \,ds \int_{a}^{s} \omega (u) \,du \biggr)^{T}R \biggl( \int_{a}^{b} \,ds \int_{a}^{s} \omega (u) \,du \biggr) \\ &- 2 \biggl( \int_{a}^{b} \,ds \int_{a}^{s} p(u)\omega (u) \,du \biggr)^{T}Rv \\ &+ \frac{4}{(b - a)^{2}} \biggl( \int_{a}^{b} \,ds \int_{a}^{s} p(u) \,du \biggr) \biggl( \int_{a}^{b} \,ds \int_{a}^{s} \omega (u) \,du \biggr)^{T}Rv. \end{aligned} $$

That is,

3 $$ \begin{aligned} [b]J(v) = {}& \int_{a}^{b} \,ds \int_{a}^{s} \omega^{T}(u)R\omega (u) \,du + \biggl( \int_{a}^{b} \,ds \int_{a}^{s} p^{2}(u) \,du \biggr) v^{T}Rv \\ &- \frac{2}{(b - a)^{2}} \biggl( \int_{a}^{b} \,ds \int_{a}^{s} \omega (u) \,du \biggr)^{T}R \biggl( \int_{a}^{b} \,ds \int_{a}^{s} \omega (u) \,du \biggr) \\ &- 2 \biggl( \int_{a}^{b} \,ds \int_{a}^{s} p(u)\omega (u) \,du \biggr)^{T}Rv \\ &+ \frac{4}{(b - a)^{2}} \biggl( \int_{a}^{b} \,ds \int_{a}^{s} p(u) \,du \biggr) \biggl( \int_{a}^{b} \,ds \int_{a}^{s} \omega (u) \,du \biggr)^{T}Rv. \end{aligned} $$

From Eq. (3), $J(v)$ has a minimum due to the fact that $J(v)$ can be taken as a quadratic function concerning vector *v* with the coefficient of the quadratic term greater than zero, i.e., $\int_{a}^{b} \,ds \int_{a}^{s} p^{2}(u) \,du > 0$.

In view of the extreme value conditions of multiple variable function, we conclude that when $\nabla J(v) = 0$, the function $J(v)$ arrives at its minimum, where ∇ denotes the nabla operator.

Namely,

$$\begin{gathered} 2R \biggl[ \int_{a}^{b} \,ds \int_{a}^{s} p^{2}(u) \,du v \\\quad{}- \biggl( \int_{a}^{b} \,ds \int_{a}^{s} p(u)\omega (u) \,du - \frac{2}{(b - a)^{2}} \int_{a}^{b} \,ds \int_{a}^{s} p(u) \,du \int_{a}^{b} \,ds \int_{a}^{s} \omega (u) \,du \biggr) \biggr] = 0.\end{gathered} $$

Solving the above equality, we can find a unique stationary point $v^{*}$:

$$\begin{aligned} v^{*} ={}& \biggl( \int_{a}^{b} \,ds \int_{a}^{s} p^{2}(u) \,du \biggr)^{ - 1} \biggl[ \int_{a}^{b} \,ds \int_{a}^{s} p(u)\omega (u) \,du \\&- \frac{2}{(b - a)^{2}} \int_{a}^{b} \,ds \int_{a}^{s} p(u) \,du \biggl( \int_{a}^{b} \,ds \int_{a}^{s} \omega (u) \,du \biggr) \biggr].\end{aligned} $$

Then

$$\begin{aligned} J\bigl(v^{*}\bigr) ={}& \int_{a}^{b} \,ds \int_{a}^{s} \biggl[\omega (u) - \frac{2}{(b - a)^{2}} \int_{a}^{b} \,ds \int_{a}^{s} \omega (r) \,dr - p(u)v^{*} \biggr]^{T} \\ &\times R\biggl[\omega (u) - \frac{2}{(b - a)^{2}} \int_{a}^{b} \,ds \int_{a}^{s} \omega (r) \,dr - p(u)v^{*} \biggr] \,du \\ ={}& \int_{a}^{b} \,ds \int_{a}^{s} \biggl[\omega (u) - \frac{2}{(b - a)^{2}} \int_{a}^{b} \,ds \int_{a}^{s} \omega (r) \,dr \biggr]^{T} \\ &\times R\biggl[\omega (u) - \frac{2}{(b - a)^{2}} \int_{a}^{b} \,ds \int_{a}^{s} \omega (r) \,dr \biggr] \,du \\ &- 2 \int_{a}^{b} \,ds \int_{a}^{s} \biggl\{ p(u)\biggl[\omega (u) - \frac{2}{(b - a)^{2}} \int_{a}^{b} \,ds \int_{a}^{s} \omega (r) \,dr \biggr] \,du\biggr\} ^{T}Rv^{*} \\ &+ \int_{a}^{b} \,ds \int_{a}^{s} p^{2}(u) \,du \bigl(v^{*}\bigr)^{T}Rv^{*} .\end{aligned} $$

In fact we have

$$\begin{gathered} - 2 \int_{a}^{b} \,ds \int_{a}^{s} \biggl\{ p(u)\biggl[\omega (u) - \frac{2}{(b - a)^{2}} \int_{a}^{b} \,ds \int_{a}^{s} \omega (r) \,dr \biggr] \,du\biggr\} ^{T}Rv^{*} \\ \quad= - 2\biggl[ \int_{a}^{b} \,ds \int_{a}^{s} p(u)\omega^{T}(u) \,du \\ \qquad{}- \frac{2}{(b - a)^{2}} \int_{a}^{b} \,ds \int_{a}^{s} p(u) \,du\biggl( \int_{a}^{b} \,ds \int_{a}^{s} \omega^{T}(u) \,du \biggr) \biggr]Rv^{*} \\ \quad= - 2 \int_{a}^{b} \,ds \int_{a}^{s} p^{2}(u) \,du \bigl(v^{*}\bigr)^{T}Rv^{*}. \end{gathered} $$

Thus

$$\begin{aligned} J\bigl(v^{*}\bigr) ={}& \int_{a}^{b} \,ds \int_{a}^{s} \omega^{T}(u)R\omega (u) \,du \\&- \frac{2}{(b - a)^{2}} \biggl( \int_{a}^{b} \,ds \int_{a}^{s} \omega (u) \,du \biggr)^{T}R \biggl( \int_{a}^{b} \,ds \int_{a}^{s} \omega (u) \,du \biggr) \\ &- \int_{a}^{b} \,ds \int_{a}^{s} p^{2}(u) \,du \bigl(v^{*}\bigr)^{T}Rv^{*}.\end{aligned} $$

Setting $p(u) = u - \frac{2a + b}{3}$, one can obtain the following equalities by basic integral calculus and integration by parts:

$$\begin{gathered} \int_{a}^{b} \,ds \int_{a}^{s} p(u) \,du = 0, \\ \int_{a}^{b} \,ds \int_{a}^{s} p^{2}(u) \,du = \frac{(b - a)^{4}}{36}, \\ \int_{a}^{b} \,ds \int_{a}^{s} p(u)\omega (u) \,du\\\quad = \frac{2}{3}(b - a)\biggl[ \int_{a}^{b} \,ds \int_{a}^{s} \omega (u) \,du - \frac{3}{b - a} \int_{a}^{b} \,ds \int_{a}^{s} \,dr \int_{a}^{r} \omega (u) \,du \biggr] .\end{gathered} $$

Additionally,

$$\begin{gathered} \int_{a}^{b} \,ds \int_{a}^{s} p^{2}(u) \,du \bigl(v^{*}\bigr)^{T}Rv^{*} \\\quad= \frac{36}{(b - a)^{4}} \biggl( \int_{a}^{b} \,ds \int_{a}^{s} p(u)\omega (u) \,du \biggr)^{T}R \biggl( \int_{a}^{b} \,ds \int_{a}^{s} p(u)\omega (u) \,du \biggr).\end{gathered} $$

Summarizing up and utilizing the above equalities yield

$$\begin{gathered} \int_{a}^{b} \,ds \int_{a}^{s} z^{T}(u)Rz(u) \,du \\ \quad= \int_{a}^{b} ds \int_{a}^{s} \omega^{T}(u)R\omega (u) \,du - \frac{2}{(b - a)^{2}} \biggl( \int_{a}^{b} ds \int_{a}^{s} \omega (u) \,du \biggr)^{T}R \biggl( \int_{a}^{b} ds \int_{a}^{s} \omega (u) \,du \biggr) \\ \qquad{}- \int_{a}^{b} \,ds \int_{a}^{s} p^{2}(u) \,du \bigl(v^{*}\bigr)^{T}Rv^{*}. \end{gathered} $$

Since $R = R^{T} > 0$, $\int_{a}^{b} \,ds \int_{a}^{s} z^{T}(u)Rz(u) \,du \ge 0$, and we conclude that inequality (1) holds.

Setting $\bar{p}(u) = u - \frac{a + 2b}{3}$ instead of $p(u) = u - \frac{2a + b}{3}$ for inequality (2) and following a similar idea to inequality (1), we can prove inequality (2). This completes the proof of the theorem. □

### Remark 1

For the sake of convenience to utilize the theorem in analyzing stability for delayed systems, inequality (1) and (2) can be equivalently rewritten as inequalities (1′) and (2′), respectively:

1′ $$\begin{aligned}& \begin{aligned}[b] &\frac{(b - a)^{2}}{2} \int_{a}^{b} \,ds \int_{a}^{s} \omega^{T}(u)R\omega (u) \,du\\ &\quad\ge \biggl( \int_{a}^{b} \,ds \int_{a}^{s} \omega (u) \,du \biggr)^{T}R \biggl( \int_{a}^{b} \,ds \int_{a}^{s} \omega (u) \,du \biggr) + 8 \Omega_{1}^{T}R \Omega_{1} ,\end{aligned} \end{aligned}$$

2′ $$\begin{aligned}& \begin{aligned}[b] &\frac{(b - a)^{2}}{2} \int_{a}^{b} \,ds \int_{s}^{b} \omega^{T}(u)R\omega (u) \,du \\&\quad\ge \biggl( \int_{a}^{b} \,ds \int_{s}^{b} \omega (u) \,du \biggr)^{T}R \biggl( \int_{a}^{b} \,ds \int_{s}^{b} \omega (u) \,du \biggr) + 8 \Omega_{2}^{T}R \Omega_{2},\end{aligned} \end{aligned}$$

where

$$\begin{gathered}\Omega_{1} = \int_{a}^{b} \,ds \int_{a}^{s} \omega (u) \,du - \frac{3}{b - a}\int_{a}^{b} \,ds \int_{a}^{s} \,dr \int_{a}^{r} \omega (u) \,du, \\ \Omega_{2} = \int_{a}^{b} \,ds \int_{s}^{b} \omega (u) \,du - \frac{3}{b - a} \int_{a}^{b} \,ds \int_{s}^{b} \,dr \int_{r}^{b} \omega (u) \,du. \end{gathered}$$

### Remark 2

Park et al. [4] also derived another Wirtinger-based double integral inequality; see [4, Corollary 1]. For comparison, we excerpt it as follows with sign change:

4 $$\begin{aligned}[b] &\frac{(b - a)^{2}}{2} \int_{a}^{b} \,ds \int_{s}^{b} \omega^{T}(u)R\omega (u) \,du\\ &\quad\ge \biggl( \int_{a}^{b} \,ds \int_{s}^{b} \omega (u) \,du \biggr)^{T}R \biggl( \int_{a}^{b} \,ds \int_{s}^{b} \omega (u) \,du \biggr) + 2 \Omega_{2}^{T}R \Omega_{2}.\end{aligned} $$

Observe that $8\Omega_{2}^{T}R \Omega_{2} > 2\Omega_{2}^{T}R \Omega_{2} \ge 0$, one easily finds that the proposed Wirtinger-type double integral inequality can deliver a tighter lower bound of the term $\int_{a}^{b} \,ds \int_{s}^{b} \omega^{T}(u) R\omega (u) \,du$ than the one in [4]. The reason lies in different derivation approaches: the former by extreme value conditions of multiple variables function; the latter mainly by the Schur complement.

### Remark 3

Apparently, by setting $\Omega_{1} = 0$, $\Omega_{2} = 0$ in Remark 1, the proposed Wirtinger-type double integral inequality reduces to Jensen’s double integral inequality [10]. Thus the theorem covers Jensen’s double integral inequality and has less conservatism than the latter owing to the fact that $8\Omega_{1}^{T}R \Omega_{1} \ge 0$, $8\Omega_{2}^{T}R \Omega_{2} \ge 0$.

When $\omega ( \cdot )$ is replaced by $\dot{x}( \cdot )$ in the theorem, we obtain the following corollary as a specialization of the theorem.

### Corollary

*For a given matrix* $R = R^{T} > 0$ *and a differentiable signal* *x* *in* $[a,b] \to \Re^{n}$ ($a < b$), *the following inequalities hold*:

5 $$\begin{aligned}& \frac{(b - a)^{2}}{2} \int_{a}^{b} \,ds \int_{a}^{s} \dot{x}^{T}(u)R\dot{x}(u) \,du \ge 3\Theta_{1}^{T}\left ( \left [ \textstyle\begin{array}{c@{\quad}c} 3 & - 8 \\ * & 24 \end{array}\displaystyle \right ] \otimes R \right )\Theta_{1}, \end{aligned}$$

6 $$\begin{aligned}& \frac{(b - a)^{2}}{2} \int_{a}^{b} \,ds \int_{s}^{b} \dot{x}^{T}(u)R\dot{x}(u) \,du \ge 3\Theta_{2}^{T}\left ( \left [ \textstyle\begin{array}{c@{\quad}c} 3 & - 8 \\ * & 24 \end{array}\displaystyle \right ] \otimes R \right )\Theta_{2}, \end{aligned}$$

*where*

$$\begin{gathered} \Theta_{1} = \left [ \textstyle\begin{array}{c} \int_{a}^{b} x(s) \,ds - (b - a)x(a) \\ \int_{a}^{b} \,ds\int_{a}^{s} x(r) \,dr - \frac{(b - a)^{2}}{2}x(a) \end{array}\displaystyle \right ],\\ \Theta_{2} = \left [ \textstyle\begin{array}{c} (b - a)x(b) - \int_{a}^{b} x(s) \,ds \\ \frac{(b - a)^{2}}{2}x(b) - \int_{a}^{b} \,ds\int_{s}^{b} x(r) \,dr \end{array}\displaystyle \right ].\end{gathered} $$

Similarly, inequalities (5) and (6) can be expressed as inequalities (5′) and (6′), respectively,

5′ $$\begin{aligned}& \begin{aligned}[b] &\frac{(b - a)^{2}}{2} \int_{a}^{b} \,ds \int_{a}^{s} \dot{x}^{T}(u)R\dot{x}(u) \,du \\ &\quad\ge \biggl( \int_{a}^{b} x(s) \,ds - (b - a)x(a) \biggr)^{T}R \biggl( \int_{a}^{b} x(s) \,ds - (b - a)x(a) \biggr) + 8 \bar{\Upsilon}_{1}^{T}R \bar{\Upsilon}_{1}, \end{aligned} \end{aligned}$$

6′ $$\begin{aligned}& \begin{aligned}[b]&\frac{(b - a)^{2}}{2} \int_{a}^{b} \,ds \int_{s}^{b} \dot{x}^{T}(u)R\dot{x}(u) \,du \\ &\quad\ge \biggl( \int_{a}^{b} x(s) \,ds - (b - a)x(b) \biggr)^{T}R \biggl( \int_{a}^{b} x(s) \,ds - (b - a)x(b) \biggr) + 8 \bar{\Upsilon}_{2}^{T}R \bar{\Upsilon}_{2}, \end{aligned} \end{aligned}$$

where

$$\begin{gathered}\bar{\Upsilon}_{1} = \frac{(b - a)}{2}x(a) + \int_{a}^{b} x(s) \,ds - \frac{3}{b - a}\int_{a}^{b} \,ds \int_{a}^{s} x(r) \,dr, \\ \bar{\Upsilon}_{2} = \frac{b - a}{2}x(b) + \int_{a}^{b} x(s) \,ds - \frac{3}{b - a} \int_{a}^{b} \,ds \int_{s}^{b} x(r) \,dr.\end{gathered} $$

## Conclusions

In this paper, a refined Wirtinger-type double integral inequality is derived on the basis of the Wirtinger-based integral inequality and the extreme value conditions of multiple variables function. The proposed inequality extends the celebrated Wirtinger-based integral inequality from a single integral to a double one. In addition, the proposed inequality refines Jensen’s double integral inequality and is less conservative compared with other double integral inequalities.

## References

[1] Seuret A., Gouaisbaut F. (2013). Wirtinger-based integral inequality: application to time-delay systems. Automatica.

[2] Seuret A., Gouaisbaut F., Fridman E. (2015). Stability of discrete-time systems with time-varying delays via a novel summation inequality. IEEE Trans. Autom. Control.

[3] Lee C.F., Yeh C.C., Hong C.H., Agarwal R.P. (2004). Lyapunov and Wirtinger inequalities. Appl. Math. Lett..

[4] Park M.J., Kwon O.M., Park J.H. (2015). Stability of time-delay systems via Wirtinger-based double integral inequality. Automatica.

[5] Liu Z., Yu J., Xu D. (2013). Vector Wirtinger-type inequality and the stability analysis of delayed neural network. Commun. Nonlinear Sci. Numer. Simul..

[6] Gu K. (2000). An integral inequality in the stability problem of time-delay systems. Proceedings of the 39th IEEE Conference on Decision Control.

[7] Park P. (1999). A delay-dependent stability criterion for systems with uncertain time-invariant delays. IEEE Trans. Autom. Control.

[8] Moon Y.S., Park P., Kwon W.H. (2001). Delay-dependent robust stabilization of uncertain state delayed systems. Int. J. Control.

[9] Sun J., Liu G.P., Chen J. (2009). Delay-dependent stability and stabilization of neutral time-delay systems. Int. J. Robust Nonlinear Control.

[10] Kwon O.M., Park M.J., Park J.H. (2013). Analysis on robust $H _{\infty}$ performance and stability for linear systems with interval time-varying state delays via some new augmented Lyapunov–Krasovskii functional. Appl. Math. Comput..

